# What constitutes competence? That depends on the task

**DOI:** 10.3109/02813432.2013.782699

**Published:** 2013-06

**Authors:** Anna Luise Kirkengen, Tor-Johan Ekeland, Linn Getz, Irene Hetlevik, Edvin Schei, Elling Ulvestad, Arne Johan Vetlesen

**Affiliations:** Professor, Department of Public Health and General Practice, Norwegian University of Science and Technology, NTNU, Trondheim, E-mail: anna.l.kirkengen@ntnu.no; Members of the ThinkTank, General Practice Research Unit, NTNU, Trondheim

General practitioners (GPs) are trained for and mandated to provide continuous healthcare to the general, unselected population in a low-threshold, local setting. This presumes as a paramount precondition that the doctor is competent as a medical helper of a large variety of people of all ages attending for an increasing variety of health-related problems or complaints. The central question as regards competence is this: How does the doctor conceptualize and understand the “entity” that enters the GP's office?

If one identifies the subject matter of medicine as diseases, carried by (more or less) average patients, then adequate medical competence involves knowledge about diseases. Thus, the wider characteristics of the diseased persons become of secondary significance. Such a framework attributes to diseases the status of essences, affecting people in the same manner, and consequently representing the core entities in medical knowledge production. This view accords with the biomedical framework of human bodies and their functions being the sum of biological mechanisms that can be unambiguously classified, and to which group-based data can be applied to explain dysfunctional states and achieve therapeutic success. If, however, one perceives that what enters the GP's office is persons, who are or feel diseased, in other words “are not at ease with themselves”, quite a different competence is demanded. Then, knowledge of the person becomes key in a double sense: first, to enable the doctor to understand how personhood in general, and this person in particular, is affected by being diseased [1]; and, even more crucially, to make the doctor capable of “reading” the present dysfunctional state as an impact on this person's embodied life [2]. The significance of knowing about a person's lifetime experiences to understand the manifestation of current or chronic sickness has been ever more solidly documented during the last two decades of multidisciplinary research [3].

According to Eric Cassell, a person is “an embodied, purposeful, thinking, feeling, emotional, reflective, relational human individual always in action, (and) responsive to meaning. Virtually all of a person's actions – volitional, habitual, instinctual, or automatic – are based on meanings” [4]. Engaging with a diseased person in an adequate manner demands, consequently, the ability to accrue an understanding of this person's unique biographical and relational context, in other words, a historical, socio-cultural, and linguistic competence. This demand precludes predefined labelling of a person's ailment in general and of reports on complex malfunction in particular. However, both clinical practice and research are currently hampered by the fact that phenomena like complex malfunction, typically termed co- or multimorbidity, are ill defined. This is a consequence of the biomedical concept of the nature of human beings and human bodies, failing to account for the fact that humans are situated beings and, as such, embedded in systems of symbols and of socio-culturally established values [5].

This complexity was recently exemplified in a study of how Nordic GPs perform detached diagnostic labelling of patients. It was based on simulated, video-recorded consultations with a common denominator: a simulated patient presenting so-called subjective health complaints (SHC) to a GP [6]. Such a presumably objective approach to human beings may in itself represent an ethically questionable reduction of both doctors and patients for several reasons [7]. First, the premise is a framework defining the subject matter of the medical encounter to be diseases or symptoms and not persons who experience illness. Second, it is questionable whether concordance in choice of labels is a potential hallmark for system quality, especially when the matter at hand is poorly defined. Third, aiming at labelling instead of understanding means failure to fulfil the central prerequisite for proper treatment. Finally, a detached method, prohibiting a mutual relationship, allows for limited insight. The study's predictable result, a considerable heterogeneity of labels, elegantly demonstrates that the construction called SHC lacks an adequate theoretical basis, and that SHC cannot be classified in a valid and reproducible manner based on the biomedical aspect alone. This fact points to the fallibility of health politics anchored in diagnostically based statistics. It also underscores the need for re-conceptualizing human beings from a medical perspective.

Human beings are embodied beings, living their lives incorporated as lived bodies. Consequently, the biomedical framework of the de-contextualized and depersonalized biological body, devoid of history and meaning, is of limited validity. Fortunately, researchers in a broad array of disciplines are currently accumulating knowledge demonstrating the impact of a person's experience on that person's body – and health [8]. In other words: the lived body, a phenomenology-derived concept subverting the mind/body dualism that still haunts biomedicine at its core, is entering into medical awareness [9]. General practitioners in the Nordic countries and, it is hoped, worldwide, may be the medical professionals who will most eagerly embrace this knowledge because it validates what most of them already know through their own accumulated professional and personal experience: human bodies are lived bodies, and lived life and health are indivisible.
